# A novel genomic region on chromosome 11 associated with fearfulness in dogs

**DOI:** 10.1038/s41398-020-0849-z

**Published:** 2020-05-28

**Authors:** R. Sarviaho, O. Hakosalo, K. Tiira, S. Sulkama, J. E. Niskanen, M. K. Hytönen, M. J. Sillanpää, H. Lohi

**Affiliations:** 1 Department of Veterinary Biosciences, University of Helsinki, 00014 Helsinki, Finland; 2 Department of Medical and Clinical Genetics, University of Helsinki, 00014 Helsinki, Finland; 3 Folkhälsan Research Center, 00290 Helsinki, Finland; 4 Equine and Small Animal Medicine, University of Helsinki, Helsinki, Finland; 5 Department of Mathematical Sciences, Biocenter Oulu and Infotech Oulu, University of Oulu, Oulu, Finland

**Keywords:** Genetics, Neuroscience

## Abstract

The complex phenotypic and genetic nature of anxieties hampers progress in unravelling their molecular etiologies. Dogs present extensive natural variation in fear and anxiety behaviour and could advance the understanding of the molecular background of behaviour due to their unique breeding history and genetic architecture. As dogs live as part of human families under constant care and monitoring, information from their behaviour and experiences are easily available. Here we have studied the genetic background of fearfulness in the Great Dane breed. Dogs were scored and categorised into cases and controls based on the results of the validated owner-completed behavioural survey. A genome-wide association study in a cohort of 124 dogs with and without socialisation as a covariate revealed a genome-wide significant locus on chromosome 11. Whole exome sequencing and whole genome sequencing revealed extensive regions of opposite homozygosity in the same locus on chromosome 11 between the cases and controls with interesting neuronal candidate genes such as *MAPK9/JNK2*, a known hippocampal regulator of anxiety. Further characterisation of the identified locus will pave the way for molecular understanding of fear in dogs and may provide a natural animal model for human anxieties.

## Introduction

Fear is an emotional state that is caused by any stimulus that is interpreted to predict or cause danger. Fear produces a behavioural reaction that aims for the survival of the animal^1,2^. However, increased and overscale fearfulness and anxiety are considered psychiatric conditions in humans and behavioural problems in dogs. In humans, anxiety disorders and specific phobias have estimated lifetime prevalences of over 15%^1^ and 7.4%, respectively^3^. While these disorders are heritable, they are genetically complex, emphasising the need of physiologically relevant animal models to facilitate the discovery of molecular etiologies^4–8^.

Domestic dogs have emerged as important large animal models of disease, morphology, and behaviour due to their naturally occurring phenotypes, unique genetic architecture and physiological similarity, and shared living environment with humans^9^. Various anxieties are commonly reported in dogs, and can dramatically impair their welfare. For example, dogs showing fear towards strangers have significantly shorter lifespans when compared to non-fearful dogs^10^. Dogs’ fearful reactions vary from withdrawing, staying close to the owner, or having a tail in a low position or placed between legs to behaviours such as barking, growling, or even biting, thus creating a serious health concern also for humans^11^. In dogs, fear can be categorised into social and non-social fearfulness. The social category includes fear of unfamiliar people and dogs while the non-social category includes fear of novel situations^12^. Our previous study suggested that fear of strange people is correlated both with fear of strange dogs and with fear of novel situations^13^. Nearly 60% of dogs showing fear of strangers were fearful in novel situations and the two traits were considered together as aspects of general fearfulness in our previous genome-wide association study (GWAS)^13,14^.

The challenge of reliably measuring behaviour and its heritable component together with the complex genetic structure underlying behaviour^15^ have slowed down the process of discovering loci or genetic variants for canine behaviour. We recently reported novel loci for canine fearfulness and noise sensitivity^14^. In addition, we have previously reported changes such as increased plasma glutamine levels in fearful dogs^16,17^. Candidate loci and genes have also been reported for example for canine fear and aggression^18^, social behaviour^19,20^, and obsessive-compulsive disorder^21,22^.

In addition to the genetic risk factors, environment has a prominent effect on fearful behaviour^23,24^. Pet dogs live their whole lives in human families and are closely monitored by their owners, enabling valid assessment of potentially important environmental factors. One factor that has a known, important role on a dog’s behaviour is the level of socialisation that the dog experiences during its puppyhood^23,25,26^. A limited amount of experiences from the surrounding world during this period is known to increase fearful behaviour in dogs^13,26^. Identification and incorporation of the environmental contributors such as socialisation into genetic analyses is important and may offer additional consolidation for a genetic signal^24^.

In this study, we have addressed the genetic background of fear of strangers in the Great Dane (GD) breed. We collected altogether 124 GD dogs with a phenotypic score using our validated behavioural survey. A GWAS and whole exome and genome sequencing revealed a novel locus on chromosome 11 that harbours interesting candidate genes.

## Materials and methods

### Study cohorts and phenotypic categorisation

A previously published and validated owner-completed behavioural survey focusing on fearfulness was used to recruit Finnish Great Dane dogs to the study^13,27^. The breed was chosen due to a large number of owner- and breeder-reported dogs that were fearful when meeting unfamiliar people.

**Fig. 1:**
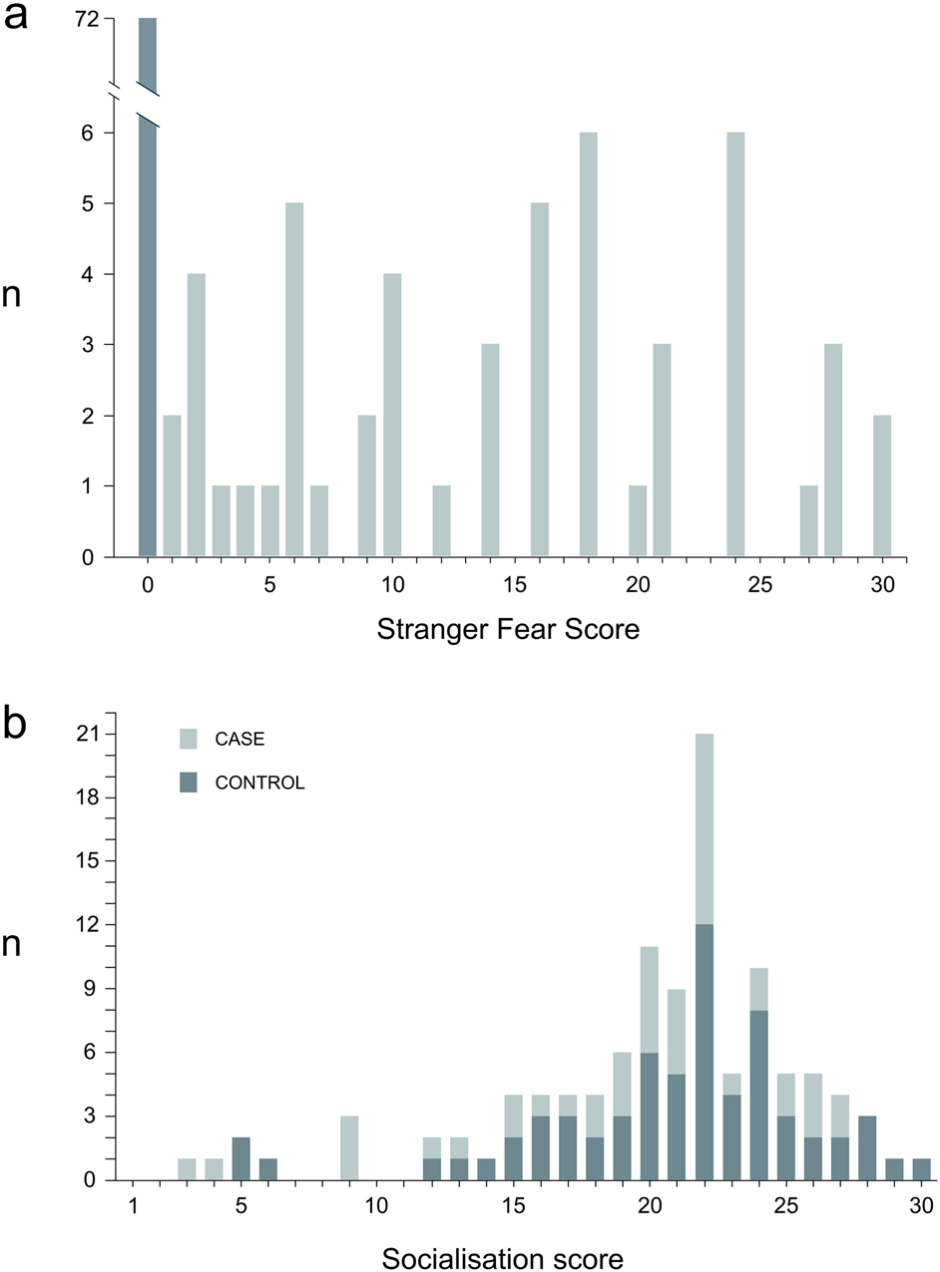
Distribution of the stranger fear and socialisation scores. The stranger fear score (**a**) was available for a total of 124 dogs. The socialisation score (**b**) was available for 110 dogs included in the stranger fear cohort. Both scores were distributed from 0 to 30 and the dogs with a stranger fear score of 0 were used as controls in the analyses.

Altogether 124 GD dogs were phenotyped (Fig. 1) using our validated behavioural questionnaire^27^. The dogs were categorised to cases and controls based on a categorical quantitative “stranger fear score” (SFS)^13^. The SFS describes the frequency and intensity of a fearful reaction towards unfamiliar people. The dogs with an SFS value of 0 showed no fearful reactions when meeting unfamiliar people and are referred to as controls. Additionally, it was required that in the questionnaire, the owners of the dogs included in the control cohort had chosen one of the following options when describing their dogs’ behaviour when meeting a novel person: “greets strangers spontaneously (if given a chance)”, “jumps, licks, is eager” or “sniffs, wags tail and is calm”. Any dog with an SFS value above 0 showed at least a mild fearful reaction and is thus referred to as a case. The SFS was calculated as follows: (sum of fearful behavioural reactions when meeting unfamiliar people, where withdrawal was multiplied by 5) × (frequency of fear reactions when meeting unfamiliar people) (Supplementary Table 1). The range of possible SFS values was from 0 to 48 with intervals of 1, with the dogs that showed the most severe and frequent fear reactions gaining the highest stranger fear (SF) scores. Avoidance was emphasised in the scoring by a factor of 5 because it is a clear, common fear reaction that is easily recognised by owners^14,28^. The case or control status of a sub-cohort (15 cases and 13 controls) of the SF-cohort was validated by a behavioural test^27^. Canine personality has been shown to be stable from 1 year onwards^29^, while it has also been suggested that fearfulness as a personality trait could be reliably observed in dogs already at the age of 6 months^30^. Thus, all control dogs were required to be over 1 year old while in the case-cohort, dogs slightly under 1 year old could be included if the dog had been through the behavioural test to confirm its status.

Based on the questionnaire answers, a socialisation score (SS) describing the extent and frequency of socialisation events during a dog’s puppyhood was also calculated. The score was used as a covariate in the genetic analyses, as socialisation is known to have a large effect on fearfulness in dogs^23,25^. The SS was calculated as a sum of socialisation event frequencies during a developmental period when the dog was between 7 weeks and 3 months of age (Supplementary Table 2). SS was previously shown to have excellent repeatability when evaluating the test–retest repeatability of the behavioural questionnaire^23^. The range of possible SS values was from 0 to 35 with intervals of 1, with the dogs that had been exposed to the highest amount of socialisation gaining the highest SSs. The SS was not available for 14 dogs with an SFS. For analysis purposes, the SS was divided into four ordered sub-categories as follows: category 1: scores from 0 to 10; category 2: scores from 10 to 20; category 3: scores from 21 to 24; and category 4: scores from 25 to 35.

According to our previous study, 57.1% of dogs that showed fearfulness when meeting a stranger were also fearful in novel situations^13^ and the two traits were studied together as general fearfulness in Sarviaho et al.^14^. In this study, we only focused on the fear of strangers. The score describing the fear in novel situations was not available for all 124 dogs included in this study (for details about the score, see Sarviaho et al.^14^). Out of the 72 controls in this study, a score that describes the level of fear they show in novel situations was available for 35 dogs. Among the 52 cases, 27 dogs had an available fear of novel situations score (Supplementary Table 3). Out of the 35 controls, three dogs showed fear in novel situations while out of the 27 cases, the equivalent number was 18. The fear in novel situations score was not used as an inclusion criterion in this study.

### Samples

Ethylenediamine tetraacetic acid blood samples were collected from 124 privately owned GD dogs for DNA. The blood samples were stored at −20 °C and genomic DNA was extracted by using a semi-automated Chemagen extraction robot (PerkinElmer Chemagen Technologie). A Qubit 3.0 Fluorometer (Thermo Fisher Scientific) and a NanoDrop ND-1000 UV/Vis Spectrophotometer were used to determine DNA concentration. Sample collection was ethically approved by the Animal Ethics Committee of State Provincial Office of Southern Finland, Hämeenlinna, Finland (ESAVI/6054/04.10.03/2012).

### Genome-wide association study

Altogether 124 GDs were genotyped using Illumina’s Canine HD (173k) single nucleotide polymorphism (SNP) arrays (Fig. 1). The data was analysed by treating SF as a quantitative trait based on the categorical SF score (Fig. 1). The initial analysis cohort included 52 cases (29 females and 23 males) and 72 controls (44 females and 28 males), where cases had an SFS between 1 and 30 and all controls had a value of 0. In the analysis cohort in which the SS was included as an environmental covariate for SFS (“socialisation cohort”), the number of cases was 44 (23 females and 21 males) and of controls 66 (40 females and 26 males). The genotype data was filtered with a SNP call rate of >90%, individual call rate of >90%, minor allele frequency of >1%, and by using a Hardy–Weinberg equilibrium test of *P* ≥ 1 × 10^−8^. After the quality control, no individual dogs were removed and 122,529 SNPs remained for analysis. Genotyping data was analysed using a quantitative trait association analysis and linear regression association analysis in PLINK^31^, and a FASTA generalised linear mixed model approach implemented in the R package GenABEL^32^. The analyses were performed using a quantitative trait approach (Fig. 1). Multidimensional scaling and quantile–quantile plots were drawn to evaluate the existence of potential confounding signals due to cryptic relatedness and population stratification in the study populations (Supplementary Fig. 1).

**Fig. 2:**
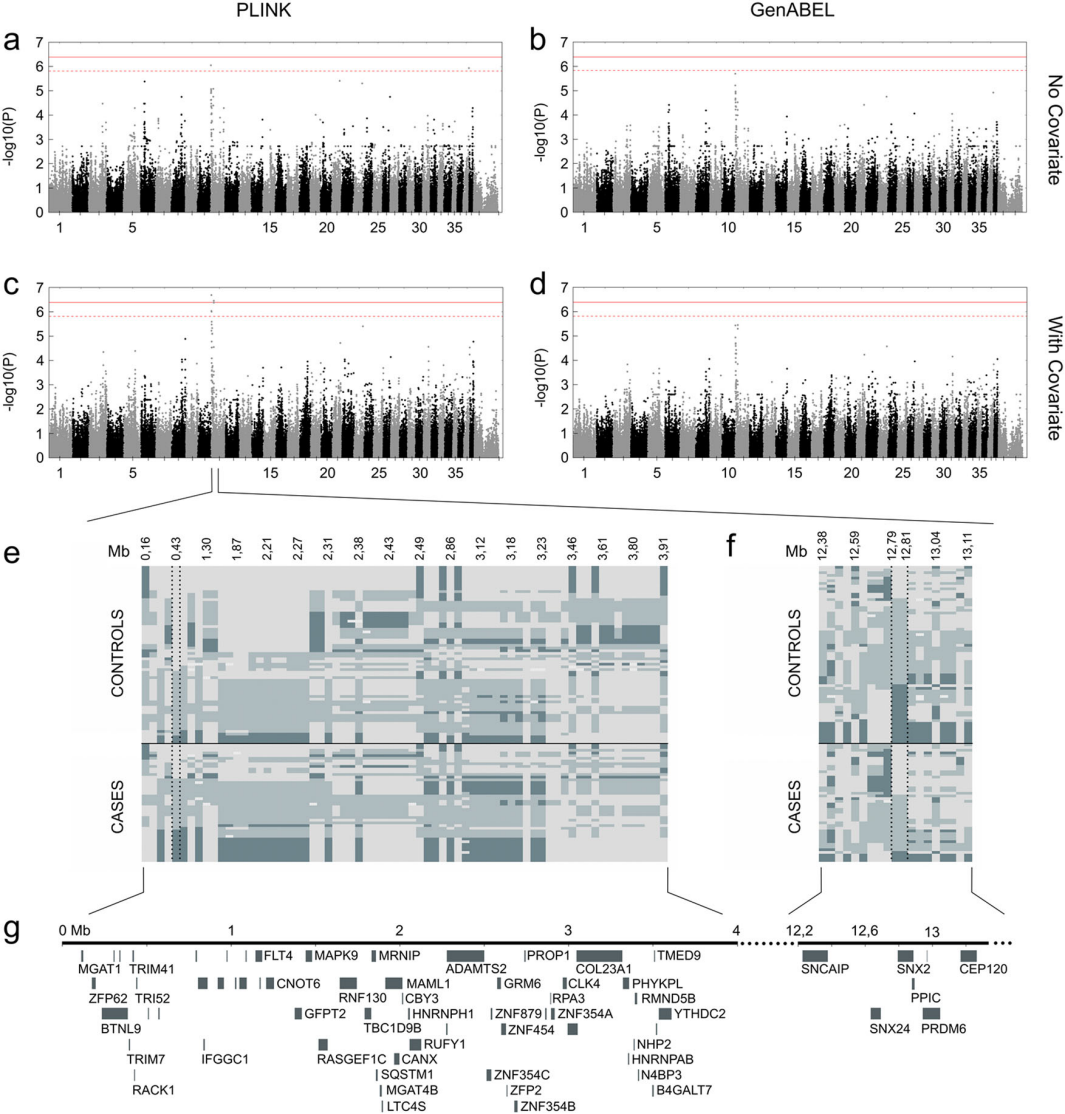
GWAS for stranger fear. **a** A Manhattan plot of a quantitative trait analysis (PLINK) illustrates best *p*-values on chromosome 11 in a genome-wide analysis with no included covariate. **b** A Manhattan plot of a mixed model association analysis (GenABEL) illustrates best *p*-values on chromosome 11 in a genome-wide analysis with no included covariate. **c** A Manhattan plot (PLINK) based on a linear regression association analysis illustrates best *p*-values on chromosome 11 in a genome-wide analysis with socialisation score as a covariate. **d** A Manhattan plot of a mixed model association analysis (GenABEL) illustrates best *p*-values on chromosome 11 in a genome-wide analysis with socialisation score as a covariate. The red intact line refers to a threshold of significance based on Bonferroni corrected values, while the red dashed line indicates a threshold of significance calculated with simpleM. **e** A genotype plot of a region on chromosome 11 from 0 to 4 Mb indicates a difference in the allelic distributions between cases and controls around the best SNP of the region at 430,548 Mb. **f** A genotype plot of a region on chromosome 11 from 12.4 to 13.1 Mb indicates a difference in the allelic distributions in the region between cases and controls around the best SNPs of the region at 12,792,149 and 12,810,167 Mb. Light grey and dark blue colours indicate the opposite homotsygote genotypes while the light blue colour indicates a heterotsygote genotype **g** The associated genomic region includes several candidate genes.

In order to adjust the data for relatedness and population stratification, we used genomic control approach with PLINK^33^ and a genomic control approach or a polygenic effect term with marker-estimated relationship matrix in GenABEL^34^. To account for multiple testing, Bonferroni correction was implemented to adjust the genome-wide significance threshold. As the Bonferroni method assumes independence between tests and is thus overly conservative for GWAS, the effective number of independent tests was estimated with simpleM^35^ and the threshold of significance calculated as described by Mikkola et al.^36^. The estimated effective number of independent tests was 34,271 for the whole cohort and 32,773 for the covariate cohort in the PLINK analyses. In the GenABEL analyses, the numbers were 34,097 and 32,678, respectively. The significance level after adjusting with Bonferroni correction was 4.08 × 10^−7^ (Fig. 2a–d). We also adjusted the significance level with the estimated number of independent tests, which resulted in a threshold of 1.46 × 10^−6^ for the PLINK analysis (Fig. 2a) and 1.47 × 10^−6^ for GenABEL (Fig. 2b) in the whole cohort analyses. For the covariate cohort, the significance level was 1.53 × 10^−6^ for both analyses (Fig. 2c, d). The linkage structure within the associated regions was determined by using PLINK’s clump command. A window size of 4000 kb was used while other settings were used as default.

### Exome and whole genome sequencing

Dog exome libraries for eight GD cases and eight GD controls were generated from standard indexed Illumina libraries using a custom Roche/Nimblegen solution-based capture library (120705_CF3_Uppsala_Broad_EZ_HX1) as previously described by Elvers et al.^37^. The 16 dogs were chosen from among the GWAS cohort based on the best SNP from GWAS (chr11:430548G>A; BICF2P368048) being from opposite haplotypes, except for one case that was a heterozygote. The dogs represented medium to severe SF phenotypes, with SF scores between 16 and 28. In addition, four cases and four controls were tested with a behavioural test as described in ref. ^27^. The exome sequencing was performed in Science for Life Laboratory (Sweden). Out of the 16 exome sequenced dogs, four GD cases and two GD controls were whole genome sequenced using the Illumina HiSeqX platform at Novogene (Novogen (HK) Company Limited, China) and the data was further processed as previously described by Hytönen and Lohi^38^. For filtering the sequencing data, Genotype Query Tools^39,40^ and our in-house analysis pipeline were used^41^. For the exome sequences, the sequencing coverage was on average 26–104× per sample and on average 105,843 variants per sample were revealed. For the whole genome sequences, the numbers were 26–33× and 6,574,191, respectively. While filtering the exome sequences, cases were filtered against the controls under a dominant model (which assumes that a variant shared by the cases is present either as heterozygous or homozygous), allowing a maximum of two cases to be wildtype homozygotes for each variant. Additionally, the controls were filtered against the cases, allowing two controls to be wildtype homozygotes for each variant. While filtering the whole genome sequences, the four cases were filtered against the two controls under a dominant model, allowing each variant to be missing from a maximum of one case. To predict the functional effects of the variants, Ensembl’s Variant Effect Predictor was used^42^. Allele frequencies were calculated for the variants by utilising 1084 exome and whole genome sequences available through our database and collaborators in the Dog Biomedical Variant Database Consortium. Conservation scores (CS) were generated with Clustal Omega^43^. Mobile element insertions (MEI) and structural variants (SV), including insertions, deletions, duplications, and intra-chromosomal re-arrangements were identified as described in Dillard et al.^44^ and by filtering the whole genome sequencing (WGS) cases against the controls under a dominant model.

Throughout the study, the CanFam 3.1 genome build was used as a reference. The short read data in BAM format for 16 WES and six WGS Great Dane samples is submitted to NCBI SRA with the BioProject ID: PRJNA630029.

### Sanger sequencing

Samples from altogether 40 GD dogs, including 15 cases (with SF scores ranging from 3 to 25) and 25 controls were used in PCR and Sanger sequencing in order to study three candidate variants (chr11:1472008G>A (SNP1292825), chr11:3390145GGGGAA>G (rs851847854) and chr11:3416964G>T (rs851521203)) identified in WES and WGS. To amplify the variant region, forward and reverse primers were designed (see Supplementary Table 4) and used together with AmpliTaq Gold 360 Mastermix (Applied Biosystems, Life Technologies) or Biotools’ DNA Polymerase. After treatment with exonuclease I and shrimp alkaline phosphatase, the sequencing reactions were performed using an ABI 3730 capillary sequencer (Applied Biosystems, Life Technologies) at the Institute for Molecular Medicine Finland (FIMM). The sequence data were analysed using Sequencher 5.1 (GeneCodes).

## Results

Altogether 124 GD were phenotyped for fearfulness towards strange people. To evaluate the intensity of the fearful reactions, a quantitative categorical “stranger fear” score (SFS) was established. Examples of a fearful and a non-fearful dog in a behavioural testing situation can be seen in Supplementary Video 1. Additionally, a quantitative categorical score evaluating the amount of socialisation (SS) during the dogs’ puppyhood was calculated and used as a covariate in the analyses. Due to the SS not being available for all 124 dogs, the covariate cohort included 110 dogs. The SFS values of cases varied between 1 and 30 while all control dogs had a value of 0. In both cohorts, the median value of SFS in cases was 16 and an average SFS value was 14.6 (Fig. 1a). The SS values varied between 3 and 30 with a median value of 21.5 and an average of 20.2 (Fig. 1b). The distribution of the SS, an environmental factor affecting SF, was rather similar between cases and controls, suggesting a genetic component for SF (Fig. 1b). For analysis purposes, the SS was further divided into four sub-categories, varying between 1 and 4, with a median of 3 and an average of 2.7. The genomic inflation factors (*λ*) were 1.097 for the whole cohort and 1.072 for the covariate cohort, suggesting moderate levels of population stratification in both cohorts (Supplementary Figs. 1 and 2).

### SF maps to chromosome 11

In order to map loci for fear of strangers, altogether 124 GD dogs were genotyped. The initial cohort included 52 cases and 72 controls, while the cohort with the SS as a covariate included 44 cases and 66 controls. The PLINK and GenABEL analyses in both cohorts revealed an association on canine chromosome 11 (Fig. 2). When including socialisation as a covariate, the PLINK analysis assuming a linear model suggested a significant association both with a Bonferroni-based threshold of significance and when the effective number of independent tests was considered (Fig. 2a), while the PLINK analysis without a covariate suggested a significant signal only with the threshold of significance calculated based on simpleM. The GenABEL analyses yielded a signal which did not reach genome-wide significance (Fig. 2b). This may be a result of a known general drawback in mixed-model approaches, causing them to lose power (by overcorrecting the structure) or possibly leading to false findings when candidate SNPs are included in the calculation of genomic relationship matrix (see, for example, Würschum and Kraft^45^).

In three of the four association analyses, the best-associated SNP was BICF2P368048 located at 430,548 bp on chromosome 11. In the GenABEL analysis including a covariate, the best-associated two SNPs spanned a region on chromosome 11 from 12,792,149 to 12,810,167 bp (Tables 1 and 2). Based on the best-associated SNPs in both analyses, two regions on chromosome 11 are associated with SF: one spanning from 0.4 Mb and one around 12.8 Mb (Tables 1 and 2).

**Table 1:** Top 10 GWAS hits in association analyses without a covariate.

PLINK (quantitative trait association analysis)			
SNP name	CHR	Position	*p*-Value (adjusted)
BICF2P368048	11	430,548	8.846e−07
BICF2S2294860	23	45,510,382	4.949e−06
BICF2P757289	11	404,385	2.385e−05
BICF2G630134982	37	29,628,498	1.164e−06
BICF2S2363579	21	41,417,917	3.89e−06
BICF2P1385199	6	20,120,837	4.158e−06
BICF2P1130863	11	12,729,090	8.292e−06
BICF2S23135525	11	1,177,541	8.494e−06
BICF2P54557	11	1,871,910	9.305e−06
BICF2P734487	11	1,848,161	1.047e−05

GenABEL			
**SNP name**	**CHR**	**Position**	***p*-Value**
BICF2P368048	11	430,548	1.99533519991599e−06
BICF2P757289	11	404,385	6.07779244096143e−06
BICF2P54557	11	1,871,910	1.07999611246649e−05
BICF2P734487	11	1,848,161	1.17827498872651e−05
BICF2G630134982	37	29,628,498	1.19426581999159e−05
TIGRP2P144053	11	1,963,139	1.30896323580011e−05
BICF2S2294860	23	45,510,382	1.76695001064685e−05
BICF2S23135525	11	1,177,541	2.08872205163928e−05
BICF2P331249	11	861,321	2.38961915938629e−05
BICF2P1130863	11	12,729,090	2.98857919837111e−05

**Table 2:** Top 10 GWAS hits in association analyses with a covariate.

PLINK (linear regression association analysis)			
SNP name	CHR	Position	*p*-Value (unadjusted)
BICF2P368048	11	430,548	2.058e−07
TIGRP2P147692	11	12,792,149	3.531e−07
BICF2P365517	11	12,810,167	3.531e−07
BICF2P1130863	11	12,729,090	4.251e−07
BICF2P757289	11	404,385	9.224e−07
BICF2S23135525	11	1,177,541	9.903e−07
BICF2P54557	11	1,871,910	2.561e−06
TIGRP2P144053	11	1,963,139	3.35e−06
BICF2S2294860	23	45,510,382	3.937e−06
BICF2S23445920	11	4,257,713	4.679e−06

GenABEL			
**SNP name**	**CHR**	**Position**	***p*-Value**
TIGRP2P147692	11	12,792,149	3.54156004567416e−06
BICF2P365517	11	12,810,167	3.54156004567416e−06
BICF2P368048	11	430,548	3.65165246465062e−06
BICF2P1130863	11	12,729,090	5.04242375996489e−06
BICF2P757289	11	404,385	1.14950271348944e−05
BICF2P54557	11	1,871,910	1.98766237111194e−05
BICF2S23135525	11	1,177,541	2.11438133432113e−05
TIGRP2P144053	11	1,963,139	2.56387893916905e−05
BICF2S2294860	23	45,510,382	2.67885122463861e−05
BICF2S23445920	11	4,257,713	3.75198642012459e−05

Genotype plots of the best-associated regions revealed differences between the cases and controls in the associated regions. Although no intact haplotypes segregating between cases and controls were seen, there seemed to be signs of haplotype structure, with many studied markers in linkage. In the region at around 12.8 Mb, the two associated SNPs were in complete linkage. To study linkage disequilibrium within both loci, PLINK’s clumping algorithm was used. For the locus at the beginning of chromosome 11, two regions of linkage were identified in the analysis without a covariate, the longer spanning from around 0.4 to 3.25 Mb (Supplementary Fig. 3a), while including the covariate resulted in only one region, spanning from roughly 0.4 to 2.4 Mb (Supplementary Fig. 3c). For the locus around 12.8 Mb, two regions were revealed in both analyses (Supplementary Fig. 3b, d). Both associated loci include several genes and the locus spanning from 0.4 Mb is largely syntenic to human 5q35.3 (Fig. 2e).

### Exome and whole genome sequencing identify extensive regions of homozygosity on chromosome 11

**Fig. 3:**
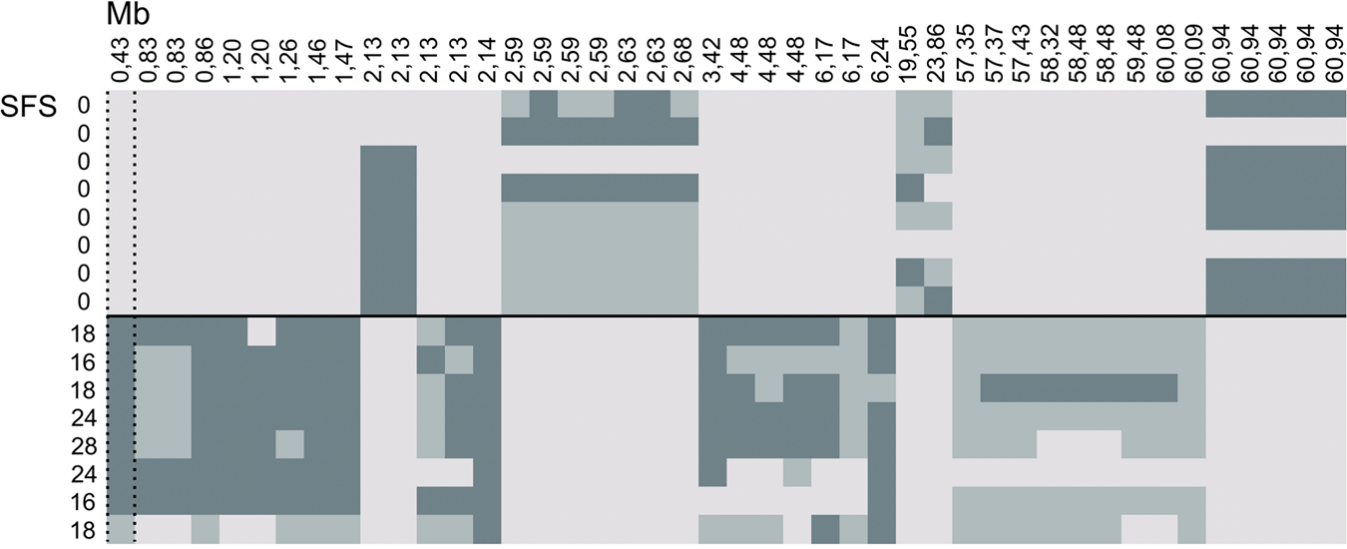
Whole exome variants between cases and controls on chromosome 11. Altogether 44 variants were left on chromosome 11 when filtering eight cases against eight controls and the controls against the cases under a dominant model and allowing a maximum of either two cases or two controls to have a deviant genotype. Light grey and dark blue colours indicate the opposite homozygote genotypes while the light blue colour indicates a heterozygote genotype. Extensive regions of opposite homozygosity are observed in the region.

Eight GD case exome sequences were filtered under a dominant model against eight GD control exome sequences. The filtering resulted in 46 variants, of which 28 laid on chromosome 11, revealing extensive regions of opposite homozygosity (Fig. 3 and Supplementary Table 5). Out of the 28 variants that were left on chromosome 11 after filtering, 13 were located between 0 and 3.5 Mb, while no variants were left around the two associated SNPs around 12 Mb.

To further study the variants on chromosome 11, four case and two control GDs were whole genome sequenced (Supplementary Table 6). After filtering with a dominant model, 73,197 variants were left, of which 4021 laid on chromosome 11. Extensive homozygosity was seen in the GWAS associated regions. Between 0 and 3.5 Mb, 478 variants segregating between cases and controls were seen, including four predicted exonic variants. Altogether 45 variants laid between 12 and 13 Mb, including two predicted exonic variants. Altogether 23 variants within the areas of interest on chromosome 11 had an allele frequency of less than 0.05, while none of these 23 variants were predicted exonic. A total of 76 variants on chromosome 11 had a CS available. Four out of the 12 variants had a CS of over 500, suggesting high conservation, and were within the regions of interest (chr11: 430548G>A (SNP1292511; rs9103113), chr11:1472008G>A (SNP1292825), chr11:3390145GGGGAA>G (rs851847854) and chr11:3416964G>T (rs851521203); Supplementary Table 6). Additionally, chr11:1472008G>A and chr11:3416964G>T were predicted exonic nonsynonymous variants within interesting neuronal candidate genes *MAPK9*/*JNK2* and *N4BP3*. While variant chr11: 430548G>A was the best SNP in GWAS analyses and was thus not followed on further, the other three variants were studied in a larger population of GDs included in GWAS. Furthermore, two ambiguous MEIs and six SVs were identified within the regions of interest (see Supplementary Table 7). One of the MEIs flagged as being ambiguous was predicted to lie within the exonic region of the immunity-related GTPase family M protein 1 gene *IRGM1*. However, the variant was not studied further due to the role of *IRGM1* in immune response. All other MEI and SVs were either intergenic or intronic and were therefore not considered for further validation.

### Validation of candidate variants in *MAPK9*, *N4BP3*, and *NHP2*

Three candidate variants were followed up by Sanger sequencing in a population 15 case and 25 control GDs. Sequencing the three variants, including two predicted exonic (in genes *MAPK9/JNK2* and *N4BP3*) and one predicted intronic variant (in the *NHP2* gene), did not reveal significant differences between cases and controls. The *p*-values were as follows: 0.79 for chr11:1472008G>A (*MAPK9/JNK2*), 0.09 for chr11:3390145GGGGAA>G (*NHP2*) and 0.95 for chr11:3416964G>T (*N4BP3*). The *p*-value was close to significance for chr11:3390145GGGGAA>G, but since the variant was not seen as homozygote in any of the tested case dogs, the difference between cases and controls did not follow the pattern seen in WGS, and thus the variant was not studied further. The results suggest that the studied three variants are likely not causal, which is in line with the fact that all three are previously known SNPs (although the allele frequencies for chr11:3390145GGGGAA>G and chr11:3416964G>T were rather low at 0.06 and 0.05; see Supplementary Table 6).

## Discussion

This study provides new insights into the genetics of fearfulness in dogs. We compared GD dogs that react fearfully when meeting a stranger with non-fearful GDs and mapped a novel susceptibility locus on chromosome 11 with an extensive level of opposite homozygosity between the case and control groups. When socialisation was included as a covariate, the association signal remained on chromosome 11 while other background signals were reduced, supporting the finding. The identified gene-rich region includes potential neuronal candidate genes, although we failed to identify robust candidate causative variants by exome or whole genome sequencing.

The GWAS, exome and WGS data suggested an SF-related locus at the beginning of chromosome 11, which is largely syntenic with human 5q35.3. The locus is rich with genes, although for many of them the available functional information is limited. The most significant SNP of the locus hits a scaffolding protein called receptor of activated protein C kinase 1, encoded by *RACK1*, alternatively named *GNB2L1*. *RACK1* seems to have a function in regulating neural development^46^, but no clear behaviour-related function for the gene has been reported.

Another candidate gene, a mitogen-activated protein (MAP) kinase gene, *MAPK9*, also known as *JNK2*, lies at 1.4 Mb on chromosome 11. MAP kinases are divided into three families, extracellular signal-regulated (ERK), p38, and c-Jun N-terminal (JNK) kinases, *MAPK9* belonging to the latter family^47^. According to studies, JNKs are involved in, for example, synaptic plasticity and brain development^48,49^ and are thought to have a role in, for example, Alzheimer’s and Parkinson’s diseases^50^. JNKs, along with the other MAPK families have also been linked to behaviour and anxiety. A study by Reinecke et al.^48^ showed some indications of changes in behaviour in an anxiety-related testing situation in *Jnk2* knock-out mice while a study by Stefanoska et al.^51^ suggested that JNKs are involved in an anxiety-modulating pathway regulated by p38α. Additionally, Mohammad et al.^52^ found that *JNK1* has a role in anxiety-related behaviour. Thus, *MAPK9* provides an interesting candidate gene for fearfulness in GDs. No segregating coding variants were found in *MAPK9* in the whole exome analyses.

At 3.4 Mb lies another candidate gene, a Fezzin family member, NEDD4-binding protein coding gene *N4BP3* that has been suggested to have a neurological function. A study by Schmeisser et al.^53^ reported that N4BP3 seems to have a role in the correct branching of neurites in developing neurons while a knock-out study by Kiem et al.^54^ indicated that the protein has a function in early anterior neural development of vertebrates. Additionally, loci harbouring Fezzin family members have been linked to neurodevelopmental disorders such as autism and schizophrenia^54–56^.

The GWAS data suggests also another associated area on chromosome 11, with a two-SNP association around 12.8 Mb (Fig. 2). The two SNPs are in full linkage and no exonic variants were left in the area after filtering (Supplementary Tables 5 and 6). The association may be spurious and needs further replication. The two associated SNPs at ~12.8 Mb on chromosome 11 hit a sorting nexin gene *SNX24*. Sorting nexins are a protein family involved in endosomal sorting and signalling^57^, some of which have been linked to neurological functions^58^.

In our previous study using the same behavioural survey, we mapped loci for noise sensitivity and general fearfulness, which included fear of strangers and fear of novel situations, in the German Shepherd breed^14^. Both associated loci were different than the novel SF locus, which suggests that SF in GDs has a separate genetic background from the general fear in German Shepherds.

In addition to our study, Zapata et al.^18^ have also studied the genetic background of stranger-oriented fear. The study identified a different association to ours, likely explained by their cross-breed setup with averaged phenotypes compared to our within-breed setup with individual behavioural scores. Thus, both studies offer a different view to the genetic background of human-directed fear. In another study, Ilska et al.^15^ studied traits called “Human and Object Fear” and “Non-owner-directed Aggression”, with the phenotypes only partly overlapping our phenotype and likely explaining different association results.

In summary, we discovered a novel fear locus with interesting candidate genes in dogs. Replicating the finding in a larger cohort, possibly narrowing down the region of interest and whole genome sequencing a larger set of cases and controls to identify candidate variants in the region will be prioritised in the future experiments. In this study, we were unable to find a risk variant for fearfulness in GDs, which likely reflects the complex genetic nature and unknown genetic mechanisms underlying the phenotype. Fear of strangers, a form of social phobia in dogs, may provide an important model for human anxieties for comparative studies.

## Supplementary information

Supplementary information

Supplementary Figure 1

Supplementary Figure 2

Supplementary Figure 3

Supplementary Table 1

Supplementary Table 2

Supplementary Table 3

Supplementary Table 4

Supplementary Table 5

Supplementary Table 6

Supplementary Table 7

Supplementary Video
