# Supplementary material for: A novel genomic region on chromosome 11 associated with fearfulness in dogs
Source: Transl Psychiatry. 2020 May 28;10:169. doi: 10.1038/s41398-020-0849-z (PMC7256038; doi:10.1038/s41398-020-0849-z)
Supplement: Supplementary file 1 — Supplementary information [file 41398_2020_849_MOESM1_ESM.docx]

**Supplementary information**

**Legends for the supplementary figures, tables and videos**

**Supplementary Figure 1.** Multidimensional scaling plot for the stranger fear cohort.

**Supplementary Figure 2.** Quantile-quantile plots for the stranger fear (SF) cohort. (**a**) q-q plot for the PLINK analysis for the SF cohort without a covariate (λ= 1.097). (**b**) q-q plot for the GenABEL analysis for the SF cohort without a covariate (λ= 1.097). (**c**) q-q plot for the PLINK analysis for the SF cohort with a covariate (λ= 1.072). (**d**) q-q plot for the GenABEL analysis for the SF cohort with a covariate (λ= 1.072).

**Supplementary Figure 3.** Linkage structure within associated regions. (a) The analysis revealed two regions of linkage between 0 and 4 Mb in the analysis without a covariate. (b) Two regions of linkage were revealed between 11 and 13.5 Mb in the analysis without a covariate. (c) One region of linkage was revealed between 0 and 4 Mb in the analysis with a covariate. (d) Two regions of linkage were revealed between 11 and 13.5 Mb in the analysis with a covariate. The smaller images beneath the main figures illustrate lengths of the proposed regions of high linkage disequilibrium. The red intact lines refer to a threshold of significance based on Bonferroni corrected values, while the red dashed lines indicate a threshold of significance calculated with simpleM. The blue vertical lines indicate the best SNPs in each analysis.

**Supplementary Table 1.** Stranger fear score. A list of behavioural survey questions addressing dogs’ reactions when meeting a novel person were scored and used in calculating the Stranger Fear score (SFS).

**Supplementary Table 2.** Socialisation score. A list of behavioural survey questions addressing how often the dog met strange women, men, children, dogs, visited a city or travelled by car or bus, when the dog was between 7 weeks and 3 months of age were scored and used in calculating the socialisation score (SS).

**Supplementary Table 3.** Stranger fear and fear of novel situation scores. The fear of novel situations score was available for 62 of the 124 dogs included in the study.

**Supplementary Table 4**. Primer pairs for sequencing three candidate variants on chromosome 11.

**Supplementary Table 5.** Exome variants on chromosome 11 after filtering eight Great Dane cases against eight Great Dane controls.

**Supplementary Table 6.** Whole genome variants and their predicted effects identified on genomic regions between 0 to 3.5 Mb and 12 to 13 Mb on chromosome 11 after filtering four Great Dane cases against two Great Dane controls.

**Supplementary Table 7**. Mobile element insertions (MEI), structural variants (SV) and their predicted effects identified on genomic regions between 0 to 3.5 Mb and 12 to 13 Mb on chromosome 11 after filtering four Great Dane cases against two Great Dane controls.

**Supplementary Video 1.** Examples of the behavioural responses of a non-fearful and fearful Great Dane when meeting a stranger during a behavioural test. The dogs that went through a behavioural test were selected based on the behavioural survey. The test was used to confirm the stranger fear status of a sub-cohort (n=28) of the initial study cohort of 124 dogs.
