# Supplementary figures and images for: A novel genomic region on chromosome 11 associated with fearfulness in dogs
Source: Transl Psychiatry. 2020 May 28;10:169. doi: 10.1038/s41398-020-0849-z (PMC7256038; doi:10.1038/s41398-020-0849-z)

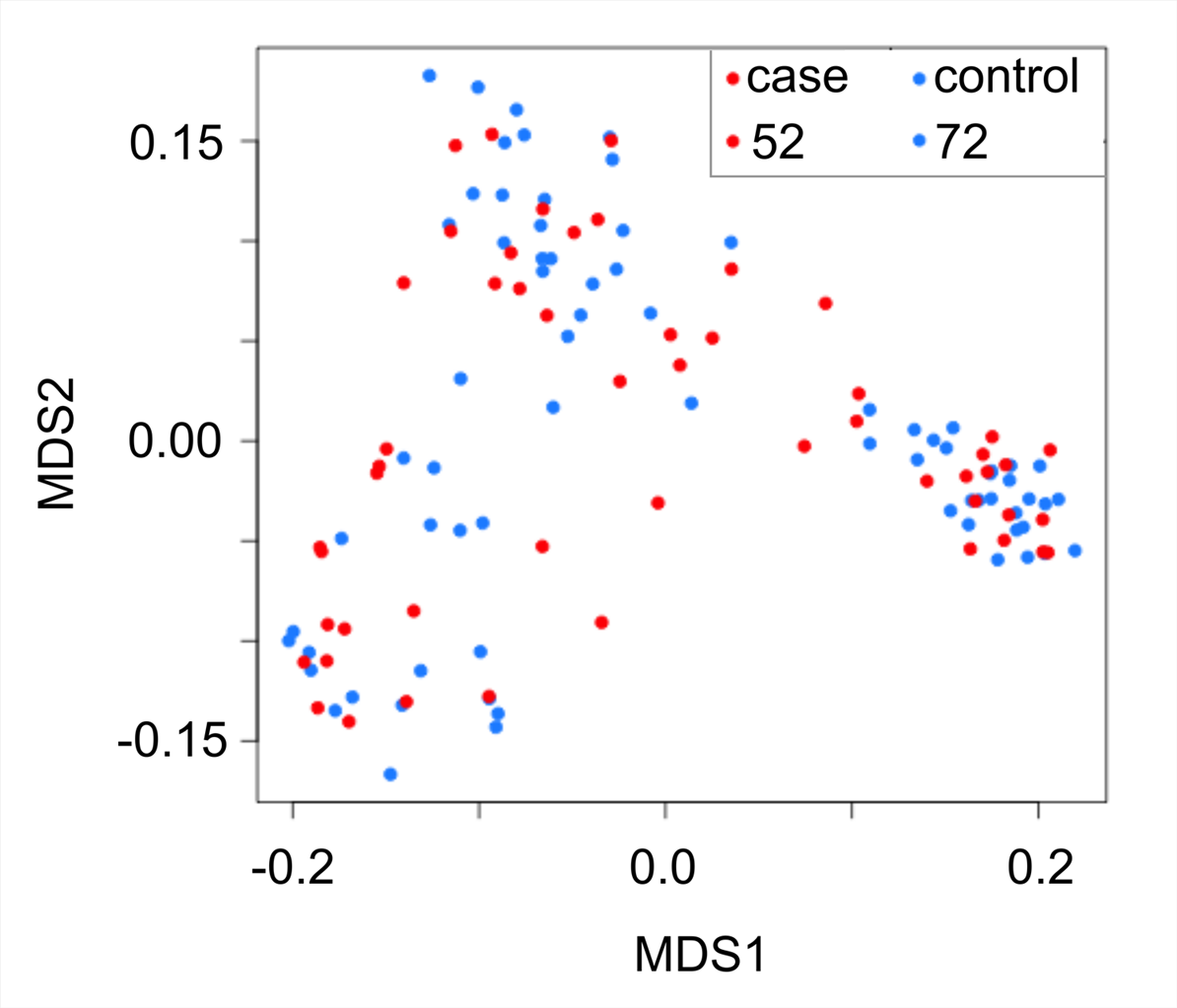

Supplement: Supplementary file 2 — Supplementary Figure 1 [file 41398_2020_849_MOESM2_ESM.png]

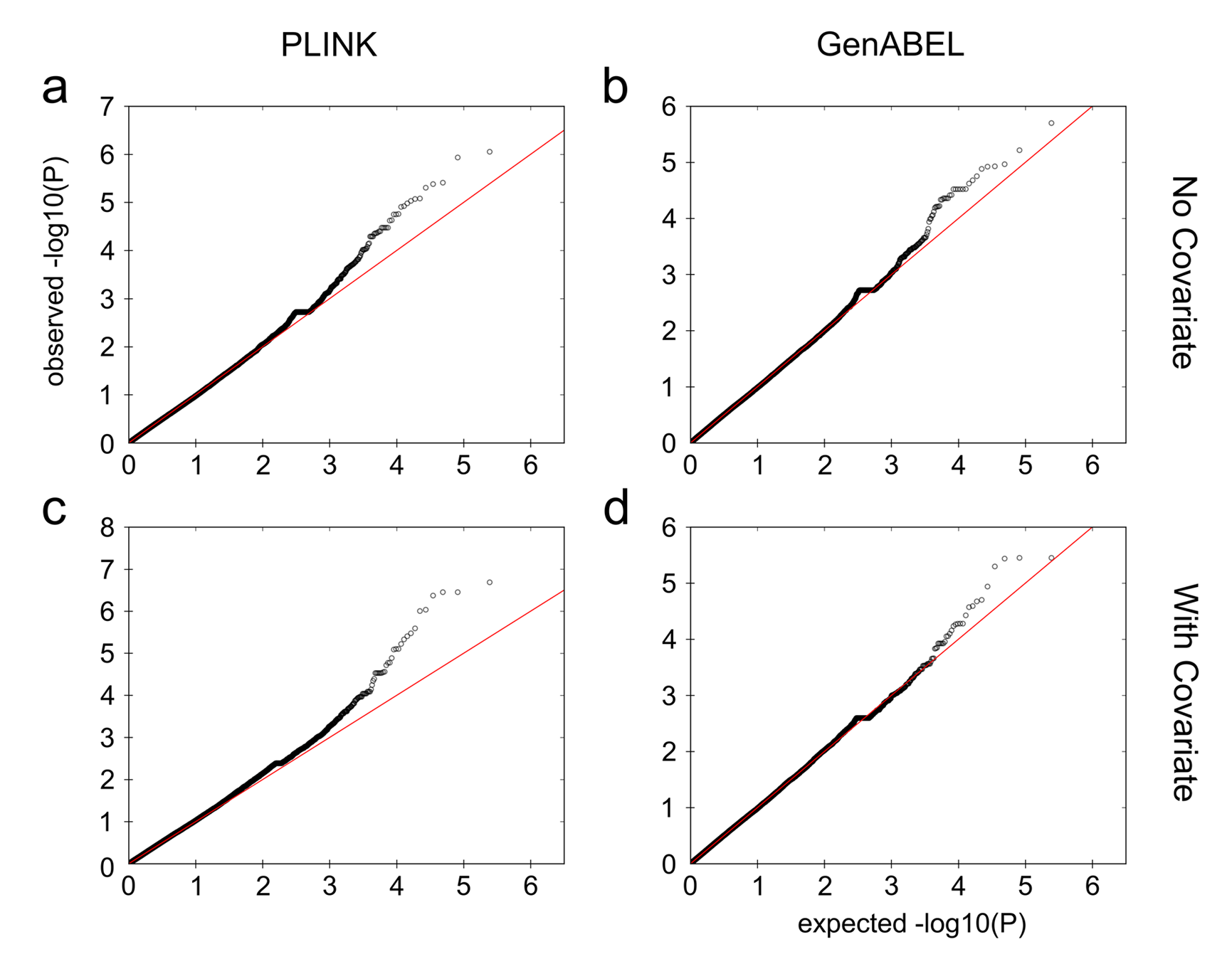

Supplement: Supplementary file 3 — Supplementary Figure 2 [file 41398_2020_849_MOESM3_ESM.png]

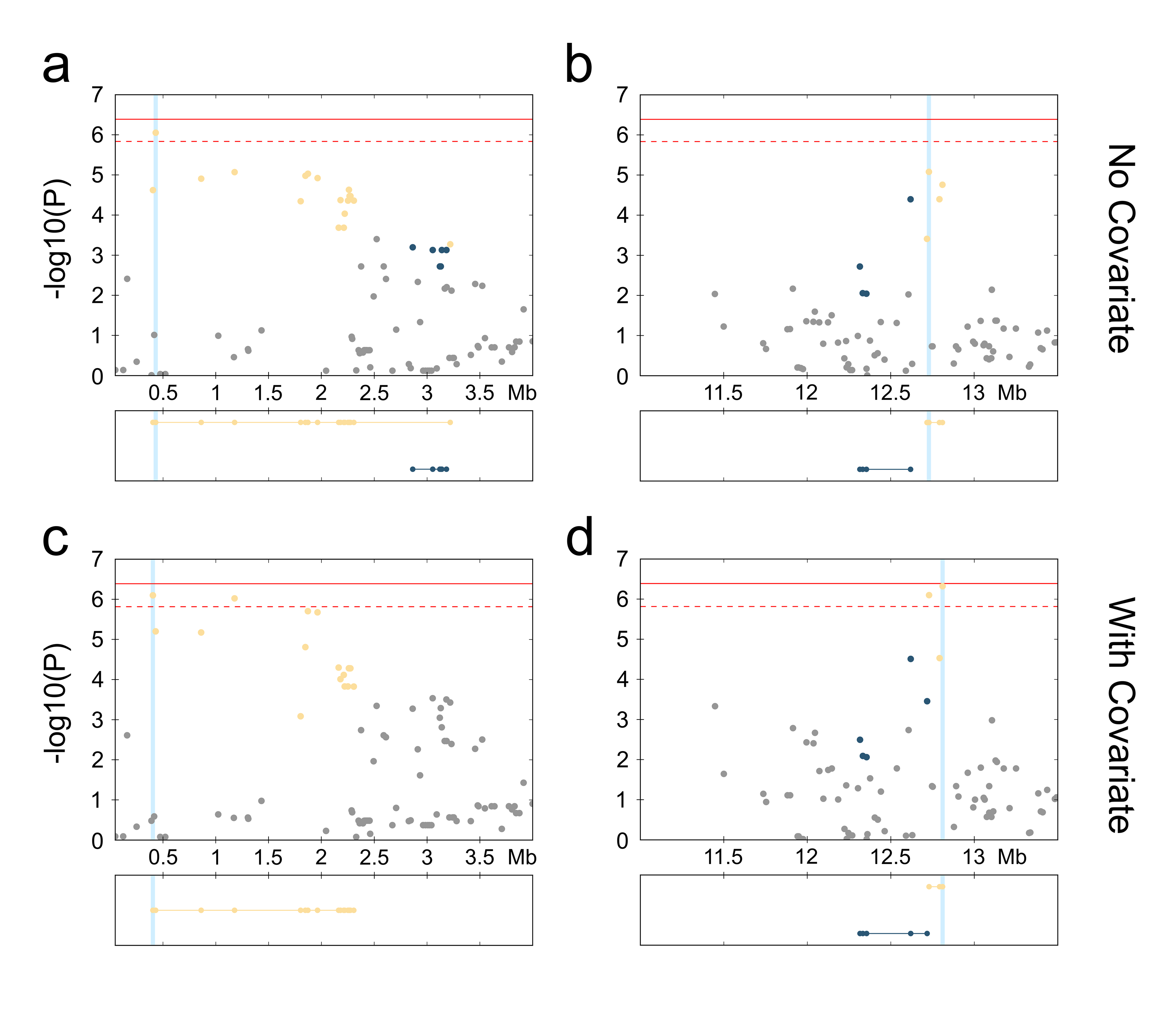

Supplement: Supplementary file 4 — Supplementary Figure 3 [file 41398_2020_849_MOESM4_ESM.png]
